# Implementation challenges of artificial intelligence (AI) in primary care: Perspectives of general practitioners in London UK

**DOI:** 10.1371/journal.pone.0314196

**Published:** 2024-11-21

**Authors:** Mohammad S. Razai, Roaa Al-bedaery, Liza Bowen, Reem Yahia, Lakshmi Chandrasekaran, Pippa Oakeshott

**Affiliations:** 1 Department of Public Health and Primary Care, University of Cambridge, Cambridge, United Kingdom; 2 St George’s School of Health and Medical Sciences, Population Health Research Institute, City St George’s University of London, London, United Kingdom; Chulalongkorn University Faculty of Medicine, THAILAND

## Abstract

**Introduction:**

Implementing artificial intelligence (AI) in healthcare, particularly in primary care settings, raises crucial questions about practical challenges and opportunities. This study aimed to explore the perspectives of general practitioners (GPs) on the impact of AI in primary care.

**Methods:**

A convenience sampling method was employed, involving a hybrid workshop with 12 GPs and 4 GP registrars. Verbal consent was obtained, and the workshop was audio recorded. Thematic analysis was conducted on the recorded data and contemporaneous notes to identify key themes.

**Results:**

The workshop took place in 2023 and included 16 GPs aged 30 to 72 of diverse backgrounds and expertise. Most (93%) were female, and five (31%) self-identified as ethnic minorities. Thematic analysis identified two key themes related to AI in primary care: the potential benefits (such as help with diagnosis and risk assessment) and the associated concerns and challenges. Sub-themes included anxieties about diagnostic accuracy, AI errors, industry influence, and overcoming integration resistance. GPs also worried about increased workload, particularly extra, unnecessary patient tests, the lack of evidence base for AI programmes or accountability of AI systems and appropriateness of AI algorithms for different population groups. Participants emphasised the importance of transparency, trust-building, and research rigour to evaluate the effectiveness and safety of AI systems in healthcare.

**Conclusion:**

The findings suggest that GPs recognise the potential of AI in primary care but raise important concerns regarding evidence base, accountability, bias and workload. The participants emphasised the need for rigorous evaluation of AI technologies. Further research and collaboration between healthcare professionals, policymakers, and technology organisations are essential to navigating these challenges and harnessing the full potential of AI.

## Introduction

The integration of artificial intelligence (AI) is highly topical, with claims that it will revolutionise medical practice and improve patient outcomes [1, 2]. AI has been defined as technologies with the ability to perform tasks that would otherwise require human intelligence [3]. However, AI in healthcare might refer to systems that go beyond predefined algorithms or templates and exhibit learning, adaptation, and contextual understanding characteristics. These systems can analyse complex data sets, derive insights, and make informed decisions autonomously or with minimal human intervention. Examples include AI-driven diagnostic algorithms, natural language processing (NLP) models, and machine learning-based predictive analytics. AI may also include tools that operate under limited circumstances, such as decision support tools, including standardised assessment templates, pop-up reminders, and risk prediction models, which operate based on predefined rules and algorithms without inherent learning capabilities. While they streamline clinical processes and aid decision-making, these tools may, at best, qualify as narrow AI programmes (i.e., perform specific tasks).

Current tools also include diagnostic imaging algorithms, predictive analytics models, clinical decision support systems such as chatbots, and healthcare robotics [1, 4]. Primary care, as the first point of contact for many patients, has the potential to benefit from AI technologies [2]. However, implementing AI in primary care requires a better understanding of its challenges, opportunities, and risks.

AI can digitalise and automate manual healthcare processes and help diagnose and detect early disease [5, 6]. Preliminary work has highlighted potential efficiency and cost-effectiveness in areas such as streamlining administrative tasks, triage, risk assessment and health coaching. The exponential expansion of medical data, encompassing electronic health records, imaging data, and electronic wearables, presents immense potential for capturing and analysing data to optimise healthcare and evaluate interventions [7].

Despite these advances, considerable risks are associated with AI use, resulting in errors that may cause harm, alongside ethical and legal implications surrounding privacy and decision-making [7]. The power of AI, particularly when combined with big data, lies in its ability to identify patterns that can be leveraged for preventive health measures and patient care [7]. However, few recent reports of GPs’ views on AI exist, and only two are from the UK [8–13]. Given the rapid advancements in AI technology, capturing up-to-date perspectives from general practitioners is crucial for ensuring its integration into primary care is effective and equitable.

To address this gap, we aimed to explore GPs’ perceptions, concerns, and practical considerations associated with adopting AI technologies in primary care in London, UK. We chose a workshop rather than smaller focus groups as this format better facilitates collaborative problem solving, practical solutions and GP education and capacity building [14].

## Methods

On 23 May 2023, we used convenience sampling to gather the perspectives of 12 GPs and four GP trainees during a hybrid workshop (in-person and online) focused on implementing AI in primary care. The workshop invitation was distributed to the mailing list of a cohort of GPs who regularly gather for a monthly academic meeting. Since the event was a workshop format, we did not engage in the recruitment and selection of individual participants. The study aimed to gain insights into GPs’ views of AI’s potential uses, impact, concerns, and challenges. Before the workshop informed verbal consent was sought from all participants, ensuring that they were aware of its purpose and their voluntary participation, and agreed to audio recording. Participants were assured of confidentiality and the anonymisation of their responses.

We used the workshop methodology [14] as it provides a conducive environment for interactive discussions, allowing participants an active role in shaping the discussion and sharing their perspectives and concerns freely without the constraints of a research setting. The group dynamic promotes collaborative problem-solving, brainstorming, and idea generation, capturing nuanced opinions and challenging existing perspectives where participants feel more involved and invested in the discussion. The facilitator (MSR) guided the discussion, exploring key topics such as the potential benefits of AI and the challenges of integrating AI into primary care and overcoming potential resistance from clinicians. Discussions followed a round-robin format, guided by questions related to each theme. The order of topics was designed to move from broad to specific issues (S1 File). Convenience sampling was chosen for its practicality in accessing general practitioners actively engaged in primary care. This approach was well-suited for this exploratory study, allowing the collection of valuable preliminary insights on an under-researched topic.

During the workshop, notes were taken to supplement the audio recording and capture the main points raised by the participants. To minimise interviewer bias, we used a standardised workshop guide. The facilitator was a trained researcher with experience of maintaining neutrality, and we cross-checked and validated the data collected through team discussion. Reflexive practices were also integrated, where the facilitator documented reflections and reviewed potential biases throughout the data analysis process.

These notes and the audio recording formed the primary data sources for analysis. Data analysis was inductive, informed by thematic analysis, as outlined by Braun and Clarke [15]. Initially, one researcher (MSR) immersed himself in the data to thoroughly familiarise himself with the content. This was followed by systematic coding of the entire dataset to generate initial codes. These initial codes were then collated into potential themes which were reviewed to ensure their consistency with the coded extracts and the broader dataset. Each theme was then clearly defined and named. The final step involved compiling the analytic narrative, integrating the data extracts, and situating the analysis within the broader existing literature.

The audio recording was transcribed by a professional service, and a sample of the data was verified for accuracy. Transcriptions of the audio recordings and notes were analysed to identify recurring themes. Two researchers conducted data coding and interpretation prior to sharing it with the broader team. Disagreements were resolved through consensus. We retained the participants’ responses in their original form to provide greater context.

In alignment with our institutional guidelines, the workshop was deemed exempt from ethics review. Additionally, we had confirmation from the NHS Health Research Authority’s algorithm that the study was classified as a service evaluation, not research. (S2 File). Although the workshop was exempt from formal ethical approval, we adhered to ethical guidelines regarding data collection, consent, and the handling of recordings.

## Results

Sixteen GPs and GP trainees aged 30 to 72 with diverse backgrounds participated in the workshop, representing various ethnicities, experiences, and expertise. Most participants were female (15/16, 93%), and five (5/16, 31%) self-identified as ethnic minorities. Participants had worked in general/family practice for 3 to 30 years and were engaged in patient-facing practices in urban and suburban settings. None worked in rural areas and none had direct experience with the application of artificial intelligence in primary healthcare for service delivery.

The following themes emerged:

1. Potential uses of AI in Primary Care
  - Improved diagnostic accuracy
  - Better patient concordance
2. Concerns and challenges with AI implementation
  - Workload
  - AI errors and accountability
  - Evidence base and evaluation of AI
  - Industry influence and financial incentives
  - Data and power imbalances in AI development
  - Overcoming resistance to AI integration

### Potential uses and impact of AI in primary care

#### Diagnosis and workload

The participants expressed interest in using AI for diagnostic purposes and acknowledged its potential to improve diagnostic pathways. However, they also raised concerns about overburdening the healthcare system with excessive testing, and the need for discussions around probabilities of disease, or abnormal results leading to further unnecessary or expensive investigations. One participant raised the issue of incorrect suggestions by the generative AI ChatGPT and emphasised that the risk of AI errors needs to be drastically reduced if it is integrated into clinical practice.

*I can see that AI could be really useful in terms of diagnostic pathways that it would need to kind of keep dipping in and dipping out; I can see as you took a patient through a diagnostic pathway, I can see it exploding investigations in patients. Though, maybe it wouldn’t. Maybe it would learn and just work, you know. Maybe we’d have ethical discussions about probabilities of various conditions that would legitimate further investigation. But I think we don’t know if it would be more. I mean if you investigate everybody for everything, then you will make every diagnosis, but have a lot of unwanted cost and tests, etc. So there’s that question around it.* [Participant 3]

*I did a trial one on ChatGPT*, *I just thought I’d test out the waters a bit*. *And I just put in “Give me a differential diagnosis for a patient vomiting blood*.*” And they said an ulcer*, *malignancy*, *and haemorrhoids*! *And I thought that was interesting because I was like*, *okay*, *well*, *that’s not right [to say haemorrhoids]*… *Obviously*, *the nuances of how it’ll be integrated into primary care is something that we need to consider*. *But in terms of diagnostics*, *I think there’s a lot to be considered in that respect*. [Participant 6]

One participant expressed enthusiasm for AI-driven solutions in healthcare that could significantly reduce clinicians’ workload. They envisioned a future where advanced software could autonomously handle entire patient consultations, capturing the nuances of decision-making and seamlessly integrating this information into health systems without the need for manual oversight. This prospect is particularly appealing as it eliminates the time-consuming task of typing up consultations, potentially allowing clinicians to focus more on direct patient care and less on administrative duties.

*We all, as clinicians, love something that will reduce our workload substantially. If we have software in a couple of years’ time that will get the entire consultation with a patient and present it in a way that is reflective of the decision-making and uploaded on the system without any need for checking. I think that would be quite an attractive prospect—not having to type the consultation.* [Participant 1]

#### Patient concordance

One participant asked about the potential use of AI in improving patient concordance but expressed scepticism about its effectiveness, given the complexity of many medication regimens.

*I can see that with something like diabetes, you know, where we’ve got a huge issue of a concordance in the population., I just wonder what AI has got to bring us in terms of concordance with increasing polypharmacy that has marginal gains for patients.* [Participant 3]

AI could potentially improve patient concordance through advanced medication management systems. For example, AI can create personalised medication schedules and reminders for patients with complex treatment regimens, like those for managing diabetes and hypertension. It can use predictive analytics to identify patterns of non-adherence and provide targeted interventions such as additional reminders or educational content. AI-powered virtual assistants can answer patient queries in real time, reducing confusion and increasing adherence.

### Concerns and challenges with AI implementation

#### Maintaining clinical skills and preventing AI errors

Participants discussed integrating AI into clinical decision-making and the importance of maintaining clinical skills while leveraging AI’s potential. One participant expressed concerns about the accuracy of AI algorithms and maintaining complex clinical skills. This is in the context of AI making diagnoses and developing management plans, as opposed to risk prediction tools that help clinicians make decisions and recommend specific management plans.

*Thinking forwards, we can only maintain clinical decision skills if we’re maintaining our skills at doing that, and with that next generation coming through, we’re going to be thinking and dependent on AI algorithms. A lot of their diagnostics are all going to be machine-driven…The AI is only as good as the information that’s put into it, which is why ChatGPT comes up with what’s on the web, but the web may not be accurate. There’s no transparency to know where that data is coming from. And then we’re gonna have clinicians. If we’re not managing complex, multiple comorbidities we deskill really quickly. And so then, how are we going to maintain that professional skills to be able to moderate the accuracy of AI?* [Participant 9]

Some cautioned that mastering simple skills first is essential for learning complex skills in clinical practice. Clinicians need a foundation of experience before relying heavily on AI. They also emphasised the need to integrate AI into medical training.

#### Accountability and responsibility for AI errors

Participants raised the issue of accountability with AI use, with discussions on who would be responsible if an AI algorithm fails to detect a diagnosis or makes an error. One participant used "C the Signs" as an illustrative example. It is a tool designed to assist healthcare professionals in the UK in the early detection of cancer by using an algorithm to analyse a combination of patient symptoms and risk factors to identify potential cancer cases early.

*We use “C the Signs”. I think, AI based system, and it would give you a percentage accuracy of certain cancers that you should be worried about. And I found that not helpful at all. Because I remember thinking they have taken away the responsibility for me, but actually, no, they haven’t because we’ve still got a responsibility to act on what they’ve told me. Do I now need to do CA125 and an ultrasound [to diagnose ovarian cancer]? Do I need to do everything that they’ve told me to do?* [Participant 9]

*So you know*, *if you go through an AI algorithm for treating your hypertension and you happen to be the one person with renal artery stenosis*. *And they have a stroke*, *who’s accountable for not having picked up that diagnosis*? *Is it the owner of the software or is it the doctor in charge*? [Participant 2]

*It’s back to liability*. *If technology organisations want to carry zero risk*, *so they’re gonna put every differential diagnosis out there and go*: *“Right over to you*!*” And I think*, *as clinicians*, *that’s a very uncomfortable place for us to be because we don’t live in a world where we ever get to zero risk*. *We carry risk*. [Participant 7]

#### Evidence base and evaluation of AI effectiveness

Participants expressed concerns regarding the evidence base for implementing AI in primary care, focusing particularly on the issues of evidence base, accountability, and effective evaluation. One participant highlighted ‘anxiety’ about the rapid adoption of AI technologies without sufficient evidence of their effectiveness. The participant feared that AI applications will "mushroom" and be "taken up" before their efficacy is adequately determined. This scenario presents a risk of integrating ineffective technologies into clinical practice. The mention of accountability reflects a concern about who will ensure that AI technologies are effective and appropriately evaluated before they become embedded in everyday clinical activities. The participant worried that AI might “come in through the backdoor" because of a general perception that AI is inherently beneficial. This concern suggests a fear that the novelty and appeal of AI could lead to a relaxation of the usual rigorous standards applied to new medical technologies. The call to "keep our research hats on" emphasised the need for ongoing scrutiny and research to ensure that AI tools are substantiated by solid evidence before they are widely adopted.

*My anxiety is about the evidence base. I mean, a lot of this will happen, and it will mushroom, and we will be taking it up, and it will become standard. And we won’t really know if [it is effective]. It goes back to your accountability, doesn’t it? There’s not going to be time for people to evaluate things and see whether they are actually effective. And you know that that worries me a bit, that we will end up taking on a lot of stuff. Much of it will be effective. But a lot of it might not be. And we won’t know necessarily when to shed it.* [Participant 4]

*It should be like other technologies that do need to have an evidence base*. *And we’re quite rigorous now about making sure*. *But I’m worried that this [AI] is going to come in through the backdoor*. *And nobody’s going to think it needs evidence because it’s AI*, *because it’s a good thing*, *or whatever*. *I think we need to keep our research hats on*. [Participant 4]

#### Influence of industry and policymakers

Participants raised concerns about the AI industry’s influence and prioritising commercial interests. One participant expressed apprehension about the industry’s disregard for a solid evidence base when pushing AI technologies. The concern extended to the industry’s significant influence over policymakers and decision-makers in the USA and the UK, whom they describe as often being naive. This suggests that these policymakers are susceptible to ambitious claims that AI will revolutionise society, partially driven by a competitive international landscape where nations like China are rapidly advancing in technology. The participant worried that this dynamic leads to hasty adoption of technologies without sufficient scrutiny of their effectiveness and safety, potentially compromising patient care for the sake of staying at the forefront of technological innovation.

*The major concern is the industry. They don’t care very much about the evidence base. They have a huge amount of not only investment but also sway over the decision-makers and policymakers in the US and the UK because policymakers are extremely naïve, most of them. They hear someone say that this [AI] is “going to change the whole society in fundamental ways. And we need to do that, and the Chinese are doing it and we are left behind”.* [Participant 2]

Another participant highlighted the critical need for balanced accuracy in diagnostics, reflecting a broader issue where commercial interests could skew medical practices away from optimal patient outcomes in the context of AI-driven diagnostics. For example, a concern was that private companies might overly promote their AI tools’ sensitivity (the ability to detect cases) while neglecting specificity (the ability to dismiss non-cases), which can lead to a high rate of false positives. This imbalance can burden the healthcare system with unnecessary treatments, increasing patient anxiety and healthcare costs.

*We teach the medical students about sensitivity and specificity. And you can imagine that the private companies will be really driving home how sensitive their approach is, and specificity won’t get a look in. But actually, that’s so important in terms of workload and patient anxiety, all of that. But it’s a much harder thing to talk about being honest about the importance of not over investigating things.* [Participant 7]

#### Financial incentives and lack of control

One participant reflected on a broader debate about the role of commercial interests in shaping healthcare practices and technologies. The fear that financial incentives might lead to suboptimal or harmful healthcare practices was palpable and called for careful consideration of how AI technologies are implemented and governed.

*I think once you start talking about profit and saving a lot of money or financial incentives, that’s a very big distraction. Because it almost feels like we have no say in this as researchers and clinicians. Something will happen. And then, we will need to find ways to adapt. And I think that’s quite a dangerous place to be.* [Participant 5]

#### Data and power imbalances in AI development and equity

One participant raised the issue of datasets used in AI decision-making and questioned their representativeness and the influence of power imbalances on data production. They pointed out that data are often assumed to reflect global populations but might not be truly inclusive or unbiased, raising the risk that AI technologies could exacerbate existing healthcare inequalities.

*The datasets being used to make all these decisions and perform tasks. Who is producing all this data that we think is representative of the world, and of the world wide web and of populations? It’s actually not true. There are a lot of power imbalances and agendas that affect AI.* [Participant 11]

*The AI technologies that are being developed*, *are these going to be relevant to everyone in healthcare in terms of race and representing certain countries over others*? *And does that mean that inequality in healthcare and global health would be just kind of propagated further*? [Participant 11]

#### Workload generation and healthcare infrastructure

The participants raised concerns about the practicality of implementing AI in the healthcare system. They discussed the workload and resource implications of integrating AI algorithms into clinical practice and the potential strain on the healthcare infrastructure. For example, the task of reviewing the algorithm-generated reports can increase the GPs workload considerably.

*One project I worked on recently which is genomics in primary care. And this one was about how you can use algorithms to pick up rare diseases and genetic diseases based on coded data in primary care. And that can seem very powerful and life-changing for patients. But one of the feedbacks I got later on from doctors going through the reports that they got from the company that runs the algorithm, was that the amount of work is just not doable.* [Participant 5]

*It’s going to be hugely disruptive*, *isn’t it*? *It’s just going to chug along*, *and just little different bits get tweaked as we go through the next 10 years*. *But I feel quite mind blown*. *[Participant 2]*

Participants also highlighted how diagnostic chatbots impact workload and risk management. The chatbots generate appropriate lists of differentials, but while clinicians mentally rule out certain possibilities, the AI verbally communicates the entire list to the patient. This creates a need for additional clinician support to manage the patient’s understanding of the diagnostic differentials and their associated risks. Primary care clinicians often manage risk without extensive testing, but this is not the case with AI-based systems. The issue of liability and responsibility is raised as technology organisations aim for zero risk by presenting all possible differentials to clinicians. This contrasts with the reality that clinicians operate in a world of inherent risk.

*I found all these different diagnostic chatbots. And it gives you a list of diagnoses. What it does is it does generate an appropriate list of differentials. But what it doesn’t do is when you use it in front of a patient, most of those differentials, you are mentally ruling things out, but you don’t tell the patient everything that’s on the list. Whereas obviously, AI does. So, in every patient with fever, leukaemia comes up on the list. … once you’ve given somebody a percentage risk of cancer, then that person wants that risk to be zero… What you’re doing as a clinician is you’re ruling those things out without an investigation and carrying a level of risk.* [Participant 7]

*It can break the health service*. *Yeah*, *really*, *you know*. *If it says you’ve got a bit of indigestion*, *and everybody has got to have six tests*. *It could literally break the health service*. *[Participant 3]*

#### Overcoming resistance to AI integration

The participants emphasised the need for cautious and thoughtful implementation of AI in healthcare. Starting with smaller applications, providing training and development opportunities for clinicians, ensuring transparency in development, and fostering confidence in AI were crucial factors in successful integration.

*So perhaps starting with small things like a UTI [Urinary Tract Infection] pathway might be the way to recognise its potential merits and then [go] bigger slowly. I think we probably just don’t know enough about what it would look like.* [Participant 12]

*I think training and education*. *I mean*, *it’s giving people enough time away from the coalface of delivering healthcare to really think about it*, *practice it and try it and build confidence in it*. *So*, *funding training and development time for clinicians is really important*. [Participant 13]

*It’s about having confidence about who’s involved in the development and the leadership*, *and I think a much more transparent conversation because I think if we’ve got confidence that senior and experienced clinicians and researchers are involved at the development stage*, *then I think people will be much more confident about being involved in the delivery*. [Participant 7]

## Discussion

### Summary of main findings

The thematic analysis revealed a range of understanding and opinions regarding implementing AI in primary care. While acknowledging the potential for improved diagnostics and efficiency, the participants also raised concerns related to patient care, increased workload, evidence-based practice, accountability, transparency, bias and lack of representativeness, and profit-driven motives of the industry. The GPs emphasised the need for rigorous evaluation of AI systems to ensure their effectiveness and safety. Additionally, the issue of accountability arose, with questions about who would be responsible for any adverse outcomes resulting from AI decisions. The GPs highlighted the potential for greatly increased workload associated with AI-generated algorithms and emphasised the need for funding and training to further integrate AI into primary care.

### Strengths and limitations

To our knowledge, this is the first study focused on GPs’ perspectives about practical challenges to AI implementation in London. We included a diverse group of GPs, representing various ethnicities, experiences and expertise, and a GP who is exploring the use of a genomic AI package in primary care. The study is highly topical and sheds light on the practical considerations, perceived benefits and potential concerns surrounding AI adoption by capturing the insights of GPs who play a crucial role in delivering primary care services.

The main limitation is that this was a small study, and the convenience sampling method limits the generalisability of the findings to other GP populations. Despite a diverse group of London GPs there may be missing voices. Additionally, the study focused on a single workshop which may not capture the full range of perspectives on the fast-moving topic of AI implementation in primary care. There is also a risk of interviewer bias. Reflexivity was integral to our study, particularly in recognising and addressing how our pre-existing beliefs and experiences with AI might have shaped our analysis and interpretation of the data. As researchers, we acknowledge our collective enthusiasm about the potential of AI to transform primary care, which may have inclined us to view the data with a positive bias. To mitigate this, we engaged in regular reflexive discussions throughout the research process, scrutinising how our perspectives influenced our coding and theme development. For instance, our prior stance led us to initially focus on the potential benefits of AI, potentially overlooking participants’ scepticism and concerns. By actively questioning our assumptions and seeking alternative interpretations, we aimed to present a balanced analysis. We also maintained a reflexive journal to document our thought processes and decisions, fostering transparency and critical self-awareness. This reflexive practice helped us to acknowledge and address our biases, ultimately striving for a more nuanced and objective interpretation of the results.

### Findings in light of other evidence

Previous studies highlighted GPs’ scepticism and limited expectations regarding AI in primary care [12]. They emphasised the importance of preserving patient-centred care and the irreplaceability of human skills like empathy, intuition, communication, and clinical reasoning. However, GPs viewed AI more positively when it came to workload reduction and patient safety [12].

Our findings differ from previous studies as they focus on practical opportunities and challenges in AI implementation rather than existential anxiety or threats to the patient-doctor relationship [11]. However, we found shared concerns about diagnostic bias, overreliance on technology, and neglecting clinicians’ expertise. Additionally, concerns about unnecessary procedures and increased workload driven by AI recommendations align with prior research. [11] As in this study, establishing trust in AI was identified as a crucial facilitator for its adoption [16].

### Implications for research and practice

The findings of this study will contribute to a better understanding of implementation challenges and opportunities of AI in primary healthcare and provide valuable insights for policymakers, healthcare organisations, and AI developers. Stakeholders must gain the trust of GPs by ensuring that AI technologies are evidence-based, improve patient care, and do not add to the workload of an already overstretched service. There is also a need for empirical studies to establish an evidence base for AI technologies in primary care. AI’s relevance to primary care lies in its potential to enhance diagnostics, streamline administrative tasks, and improve patient engagement. However, unique challenges include data privacy concerns, the need for user-friendly interfaces, and ensuring equitable access and outcomes across different patient populations. Future studies of AI systems should focus on evaluating their effectiveness, safety, and impact on GP workload and patient outcomes. Additionally, accountability, liability, trust and transparency should be addressed to ensure patient safety, optimise healthcare outcomes, and leverage the potential of AI technologies in primary care.

## Supporting information

S1 Checklist Human participants research checklist. (DOCX)

S1 File Discussion topic guide. (DOCX)

S2 File MRC and NHS HRA criteria. (PDF)

## References

[1] Kumar P, Sharma SK, Dutot V. Artificial intelligence (AI)-enabled CRM capability in healthcare: The impact on service innovation. International Journal of Information Management. 2023;69:102598.

[2] Lin SY, Mahoney MR, Sinsky CA. Ten ways artificial intelligence will transform primary care. Journal of general internal medicine. 2019;34:1626–30. doi: 10.1007/s11606-019-05035-1 31090027 PMC6667610

[3] Industrial Strategy: building a Britain fit for the future, Department for Business, Energy and Industry Strategy, Policy Paper. 2017.

[4] Singh B, Olds T, Brinsley J, Dumuid D, Virgara R, Matricciani L, et al. Systematic review and meta-analysis of the effectiveness of chatbots on lifestyle behaviours. npj Digital Medicine. 2023;6(1):118. doi: 10.1038/s41746-023-00856-1 37353578 PMC10290125

[5] Yu Z, Nguyen J, Nguyen TD, Kelly J, Mclean C, Bonnington P, et al. Early melanoma diagnosis with sequential dermoscopic images. IEEE Transactions on Medical Imaging. 2021;41(3):633–46. 10.1109/TMI.2021.3120091 34648437

[6] Bernardini M, Romeo L, Frontoni E, Amini M-R. A semi-supervised multi-task learning approach for predicting short-term kidney disease evolution. IEEE Journal of Biomedical and Health Informatics. 2021;25(10):3983–94. doi: 10.1109/JBHI.2021.3074206 33877990

[7] Ali O, Abdelbaki W, Shrestha A, Elbasi E, Alryalat MAA, Dwivedi YK. A systematic literature review of artificial intelligence in the healthcare sector: Benefits, challenges, methodologies, and functionalities. Journal of Innovation & Knowledge. 2023;8(1):100333.

[8] Ellertsson S, Loftsson H, Sigurdsson EL. Artificial intelligence in the GPs office: a retrospective study on diagnostic accuracy. Scand J Prim Health Care. 2021;39(4):448–58. doi: 10.1080/02813432.2021.1973255 34585629 PMC8725823

[9] Micocci M, Borsci S, Thakerar V, Walne S, Manshadi Y, Edridge F, et al. Attitudes towards Trusting Artificial Intelligence Insights and Factors to Prevent the Passive Adherence of GPs: A Pilot Study. J Clin Med. 2021;10(14). doi: 10.3390/jcm10143101 34300267 PMC8303875

[10] Kueper JK, Terry AL, Zwarenstein M, Lizotte DJ. Artificial intelligence and primary care research: a scoping review. The annals of family medicine. 2020;18(3):250–8. doi: 10.1370/afm.2518 32393561 PMC7213996

[11] Buck C, Doctor E, Hennrich J, Jöhnk J, Eymann T. General practitioners’ attitudes toward artificial intelligence–enabled systems: interview study. Journal of Medical Internet Research. 2022;24(1):e28916. doi: 10.2196/28916 35084342 PMC8832268

[12] Blease C, Kaptchuk TJ, Bernstein MH, Mandl KD, Halamka JD, DesRoches CM. Artificial intelligence and the future of primary care: exploratory qualitative study of UK general practitioners’ views. Journal of medical Internet research. 2019;21(3):e12802. doi: 10.2196/12802 30892270 PMC6446158

[13] Tabla S, Calafiore M, Legrand B, Descamps A, Andre C, Rochoy M, et al. Artificial Intelligence and Clinical Decision Support Systems or Automated Interpreters: What Characteristics Are Expected by French General Practitioners? Stud Health Technol Inform. 2022;290:887–91. doi: 10.3233/SHTI220207 35673146

[14] Ørngreen R, Levinsen K. Workshops as a Research Methodology. Electronic Journal of E-learning. 2017;15(1):70–81.

[15] Cooper H, Long D, Braun V, Clarke V. Thematic analysis. APA handbook of research methods in psychology, Research designs: quantitative, qualitative, neuropsychological and biological Washington DC: American Psychological. 2021.

[16] Dellermann D, Ebel P, Söllner M, Leimeister J. Hybrid Intelligence. Bus Inf Syst Eng 61: 637–643. 2019.

