# Supplementary material for: Implementation challenges of artificial intelligence (AI) in primary care: Perspectives of general practitioners in London UK
Source: PLoS One. 2024 Nov 21;19(11):e0314196. doi: 10.1371/journal.pone.0314196 (PMC11581230; doi:10.1371/journal.pone.0314196)
Supplement: S1 File — (DOCX) [file pone.0314196.s002.docx]

**S1 File Discussion Topic Guide**

**Implementation challenges of artificial intelligence (AI) in primary care: perspectives of ethnically diverse general practitioners in the UK**

**Understanding AI in Primary Care**

- **Prompt:** "What is your current understanding and perception of AI in primary care?"
- **Discussion Points:**
  - Variations in the level of AI awareness among primary care professionals
  - Real-world examples of AI applications in primary care
  - Expectations versus realities of AI integration in healthcare settings

**Potential Uses and Impact of AI**

- **Prompt:** "Identify potential uses of AI in your daily primary care practices."
- **Discussion Points:**
  - AI in diagnostic processes, patient triaging, and treatment plans
  - Impact of AI on patient outcomes and clinical decision-making
  - The role of AI in enhancing patient engagement and personalised care

**Addressing Concerns and Challenges**

- **Prompt:** "Discuss the main concerns and ethical challenges in implementing AI in primary care."
- **Discussion Points:**
  - Data privacy, security, and consent in the context of AI
  - Addressing biases and ensuring equity in AI algorithms
  - Managing the integration of AI with existing healthcare systems and workflows

**Collaborative Action Planning**

- **Prompt:** "Develop a collaborative action plan for effective AI implementation in your practice."
- **Discussion Points:**
  - Identifying key stakeholders and their roles in AI implementation
  - Strategies for training and supporting primary care staff in AI adoption.
  - Setting realistic goals and measuring the success of AI integration

**Wrap-Up and Next Steps**

- **Prompt:** "Reflect on the insights gained today and propose the next steps for AI integration in primary care."
- **Discussion Points:**
  - Collaborative strategies for ongoing learning and adaptation to AI advancements.
  - Building a network for sharing best practices and experiences in AI implementation.
